# Genetic biomarkers for PD-1/PD-L1 blockade therapy

**DOI:** 10.18632/oncoscience.328

**Published:** 2016-11-21

**Authors:** Keisuke Kataoka, Seishi Ogawa

**Affiliations:** Department of Pathology and Tumor Biology, Graduate School of Medicine, Kyoto University, Yoshida Konoe-cho, Sakyo-ku, Kyoto, Japan, 606-8501

**Keywords:** PD-L1, PD-1, cancer, immune checkpoint, biomarker

Immune checkpoint blockade therapy using antibodies against programmed cell death 1 (PD-1) and PD-1 ligand 1 (PD-L1) is revolutionizing cancer treatment (1-3). These antibodies provide long-term durable responses for patients with various types of advanced cancers, such as melanoma, non–small-cell lung cancer, kidney cancer, and Hodgkin lymphoma (1-3). Accumulating evidence suggests that these agents convey their therapeutic effects through targeting the PD-1/PD-L1 immune checkpoint to unleash anti-tumor immune responses. In turn, it is supposed that cancer cells depend critically on evading immune surveillance for their malignant growth. As immune checkpoint blockade therapy has benefited only a subset of patients, defining biomarkers that can predict therapeutic efficacy and adverse effects is of urgent importance, which is substantiated by recent approvals for PD-L1 diagnostic tests (2). Here we provide a brief overview of the recent development of genetic biomarkers for PD-1/PD-L1 blockade therapies, with special focus on *PD-L1* genetic abnormalities, including its 3′-UTR disruption.

Early studies demonstrated the potential value of immunological biomarkers, such as intratumoral lymphoid infiltrates and PD-L1 expression on tumor or infiltrating immune cells, for predicting response to PD-1/PD-L1 blockade (1, 2). However, subsequent studies have revealed a lower but significant response rate in patients with PD-L1^−^ tumors, raising questions about the utility of these immunological markers as an ideal selection criterion for PD-1/PD-L1 blockade therapy (2). Indeed, estimated from the results across 15 studies including various solid cancer types, the overall response rate to PD-1/PD-L1 blockade was 48% in patients with PD-L1^+^ tumors, in contrast to 15% in those with PD-L1^−^ tumors. In addition, especially in clinical setting, accurate measurement and scoring of PD-L1 protein expression are hampered by a variety of technical and biological pitfalls (2).

Genomics-based approaches have the potential to complement immunological biomarkers. In particular, Rizvi et al. demonstrated that a higher load of nonsynonymous mutations and neoantigens detected by whole-exome sequencing positively correlated with clinical response to an anti-PD-1 antibody (pembrolizumab) in non-small cell lung cancer (NSCLC) patients. Moreover, candidate neoantigens were experimentally validated using a high-throughput multimer screening to identify neoantigen-specific T cells. In one responder, neoantigen-specific T-cell reactivity paralleled tumor regression (4). In addition to neoantigen load, the extent of neoantigen intratumoral heterogeneity (ITH) within single tumors affects the sensitivity to immune modulation. An integrated analysis of ITH and neoantigen burden showed that the response to PD-1 blockade in patients with NSCLC was enhanced in tumors enriched for clonal neoantigens, i.e., those shared by the major tumor population, whereas cytotoxic chemotherapy-induced subclonal neoantigens, contributing to an increased mutational load, were enriched in poor responders. In addition, T cells recognizing clonal neoantigens were detectable in patients with durable clinical benefit (5).

It has been reported that several classes of mutations which can generate a large number of somatic lesions are associated with the susceptibility to PD-1/PD-L1 blockade. For instance, although advanced colorectal cancers are generally unresponsive to anti-PD-1 therapy, a subset with mismatch-repair deficiency show high somatic mutation loads and exhibit a higher response rate and improved survival. The response to anti-PD-1 therapy may not depend on tumor type, as exemplified by a similar good response in patients with mismatch repair-deficient noncolorectal cancers (2). Another example is *BRCA2* mutation, which is related to DNA repair and replication. In patients with melanoma treated with pembrolizumab, *BRCA2* mutations were enriched in those who were responsive to PD-1 blockade (6).

Besides overall mutational landscape, genomic profiling has identified a certain type of mutations which can induce PD-L1 expression and are expected to affect the response to immune checkpoint blockade therapy (2). Notably, *PTEN* deletion was shown to enhance PD-L1 expression through upregulation of the PI3K-AKT pathway in glioblastoma. Similarly, constitutively activated ALK signaling, observed in a subset of lymphomas and NSCLC, has been reported to drive PD-L1 expression through STAT3 activation. Moreover, genetically engineered mouse models of lung cancer with mutant EGFR or dual loss of LKB1 and PTEN demonstrated PD-L1 induction (2).

Genetic biomarkers may identify a small subset of patients who are likely to benefit from immune checkpoint blockade therapy (exceptional responders), from within a largely unresponsive population. Especially, genetic abnormalities in *PD-L1* itself leading to its overexpression seem to have a substantial impact on response to PD-1/PD-L1 inhibitors. This possibility is strongly supported by the impressive efficacy of anti-PD-1 therapy in Hodgkin lymphoma, in which the majority of cases have copy number gain or amplification involving *PD-L1* and/or *PD-L2* (3).

*PD-L1*-involving genetic alterations have also been reported in various other cancer types. For example, *PD-L1* amplification or copy number gain is observed in a subset of B-cell non-Hodgkin's lymphomas and stomach adenocarcinoma. Another genetic mechanism inducing PD-L1 activation reported in primary mediastinal B-cell lymphoma is utilization of an ectopic promoter caused by chromosomal translocation (1). More recently, a unique genetic mechanism for cancer immune evasion through aberrant PD-L1 expression has been reported, which is caused by 3′-UTR disruption (7). Initially identified in adult T-cell leukemia/lymphoma (8), structural variations (SVs) affecting *PD-L1* 3′-UTR were found in a wide variety of tumor histologies, including diffuse large B-cell lymphomas and stomach adenocarcinomas (7). Resulting from different types of SVs, including deletions, inversions, tandem duplications, and translocations, the 3′-UTR disruption almost always resulted in markedly elevated *PD-L1* transcripts (1). The role of these SVs was confirmed by *in vitro* and *in vivo* experiments using CRISPR/Cas9 technology, which showed that the 3′-UTR disruption can accelerate immune evasion of tumor cells (7). These genetic abnormalities, especially those in *PD-L1* itself, should be exploited as a potential genomic biomarker to select patients who are expected to achieve long-term durable response to PD-1/PD-L1 blockade therapy. The genomics-based approaches will hopefully allow for development of reliable biomarkers to better guide immune checkpoint blockade therapy for cancer patients.
